# Low-carbon economic dispatch considering integrated demand response and multistep carbon trading for multi-energy microgrid

**DOI:** 10.1038/s41598-022-10123-0

**Published:** 2022-04-13

**Authors:** Yilin Long, Yong Li, Yahui Wang, Yijia Cao, Lin Jiang, Yicheng Zhou, Youyue Deng, Yosuke Nakanishi

**Affiliations:** 1 College of Electrical and Information Engineering, Hunan University, Changsha, 410082 China; 2 Department of Electrical Engineering and Electronics, University of Liverpool, Liverpool, L693BX England; 3 Graduate School of Environment and Energy Engineering, Waseda University, Tokyo, 169-8050 Japan

**Keywords:** Energy grids and networks, Power distribution

## Abstract

With the rapid development of distributed energy resources and natural gas power generation, multi-energy microgrid (MEMG) is considered as a critical technology to increase the penetration of renewable energy and achieve the target of carbon emission reduction. Therefore, this paper proposes a low-carbon economic dispatch model for MEMG to minimize the daily operation cost by considering integrated demand response (IDR) and multistep carbon trading. Specifically, IDR operation includes shifting of shiftable electric load, adjusting of flexible thermal load and cooling load, and it is employed to decrease operation cost. Besides, the multistep carbon trading means that different carbon trading prices correspond to different carbon trading volumes, which is applied to stringently restrict carbon emission. The simulation results show that the proposed model can effectively reduce the carbon emission while greatly decrease the operation cost.

## Introduction

Application of low-carbon energy is an efficient measure to accelerate the process of carbon peak and carbon neutral. With the rapid development of renewable energy power generation and natural gas power generation, multi-energy microgrid (MEMG) is considered as a critical technology to increase the proportion of using renewable energy and achieve the target of carbon emission reduction^1^. MEMG, in which the electrical microgrid act as the backbone of the multi-vector energy system, can coordinate the supply and consumption of many kinds of energy such as cooling, thermal and electricity^2^.

Recently, some researches about the optimal operation of MEMG have been carried out at home and abroad. Zhang et al.^3^ proposed a robust coordinated operation approach which coordinates multiple devices in different timescales to minimize the operating costs. A multi-objective optimal dispatching model for a grid-connected microgrid considering wind power forecasting probability was established by Sun et al.^4^. Liu et al.^5^ proposed a day-ahead optimal operation strategy by utilizing distributed energy resources based on the framework of the interconnected multi-energy system to address the negative impacts of intermittent renewable energy sources. Zhang et al.^6^ proposed a model of industrial production process by dividing the process into different adjustable steps, including continuous subtask, discrete subtask, and storage subtask, considering the coupling between the production process and energy demands. The above studies only consider the overall economic cost of the system and ignore the additional environmental cost caused by carbon emission.

In order to reduce the carbon emission of the energy system, carbon trading is considered to be an effective way to improve low-carbon environmental protection^7^. Carbon trading is a trading mechanism that controls carbon emissions by establishing legal carbon emission rights and allowing them to be bought and sold^8^. Wang et al.^9^ proposed a low-carbon economy operation model of integrated energy system (IES) considering life cycle assessment energy chain and carbon trading mechanism, which can effectively promote the low-carbon development of IES. The carbon trading mechanism was applied to the IES planning model by Qiu et al.^10^, which alleviates the contradiction between the economy and low carbon of low carbon energy generation. Wei et al.^11^ proposed a low-carbon economy operation model of power-gas interconnection IES and analyzed the impact of carbon trading price on system operation. A decentralized scheduling model for multi-region IES considering carbon trading cost is proposed by Zhai et al.^12^. The carbon trading cost models of above studies all employ the unified carbon trading cost model that does not divide the carbon emission amount into different sections. The carbon trading cost model is improved and a multistep carbon trading cost model is proposed in this paper, which can more stringently constraint the carbon emission. However, multistep carbon trading model leads to cost increasing in the result of carbon trading price rising as carbon emission increasing.

Integrated demand response (IDR) refers to the autonomous response behavior in which users adjust the demands of different energy sources to achieve the cost saving goal^13,14^, which can decrease the energy cost. Therefore, IDR is taken into consideration to counteract cost increase on account of multistep carbon trading. This paper proposes a low-carbon economic scheduling model of MEMG considering IDR and multistep carbon trading to improve economy and realize environmental protection. Compared with the existing research works, the innovation and contribution of this paper are as follows:

1. An IDR model including shiftable and interruptible loads, shiftable but uninterruptible loads, flexible thermal and cooling loads is proposed. Meanwhile, a new modeling method to make shiftable but uninterruptible electric loads model linear is carried out.
2. The multistep carbon trading mechanism is proposed to stringently constrain the carbon emission of MEMG.
3. Through case analysis, it is verified that the proposed model is economic and eco-friendly. The sensitivity analysis of the parameter *v* (the interval length of carbon emission) and the rated power of gas turbine are carried out.

The rest of the paper is organized as follows. Section 2 presents the architecture of MEMG. The low-carbon economic dispatch model of MEMG is shown in Sect. 3, which include the introduction of the proposed optimal dispatch framework of MEMG, multistep carbon trading model and IDR model. In Sect. 4, the simulation results and analysis of different cases are shown as well as the sensitivity analysis. Section 5 is the conclusion of this paper.

## The architecture of MEMG

**Figure 1:**
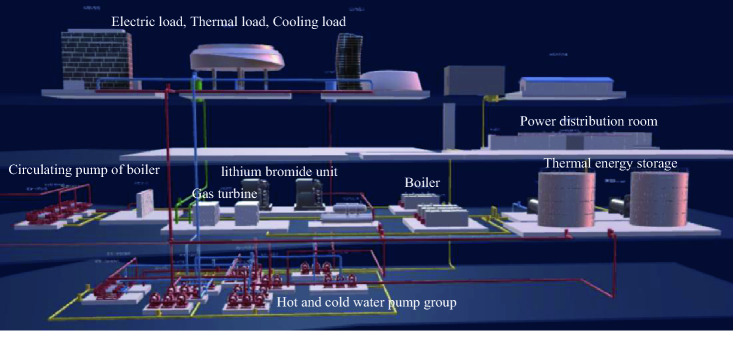
The energy station system in central south of China.

**Figure 2:**
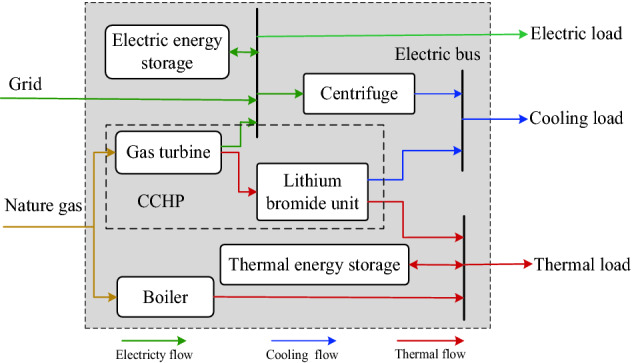
The MEMG architecture.

The MEMG architecture constructed in this paper refers to an actual energy station that can satisfy electric demand, thermal demand and cooling demand. The energy station system is located in central south of China, as shown in Fig. 1. The MEMG architecture is shown in Fig. 2. The energy supply side includes external grid and natural gas. The energy conversion appliances are equipped with combined cooling, heat and power generation (CCHP), boiler and centrifuge. As for load side, there are storage devices, electric load, cooling load and thermal load.

### CCHP

The CCHP that consists of gas turbine and lithium bromide unit can convert nature gas to electric energy, thermal energy and cooling energy. The gas turbine can produce high temperature fuel gas while output electric energy. And the lithium bromide unit can use the high temperature fuel gas to generate cooling or thermal energy, which makes the waste thermal be fully utilized and leads to little thermal loss.

Equation (1) represents the calculation method of electric energy, which equals to the product of power generation efficiency of gas turbine (${\eta }_{gen,e}$), calorific value of natural gas (*λ*_*gas*_) and consumed natural gas (*n*_*gen*_(*t*)). *λ*_*gas*_ is equal to 9.97 kWh/m^3^. *P*_*gen*_(*t*) represents the electricity generated by gas turbine*. P*_*gen,rated*_ is the rated power of gas turbine.

1 $$P_{gen} (t) = \eta_{gen,e} \cdot \lambda_{gas} \cdot n_{gen} (t){/}\Delta t$$

2 $$0 \le P_{gen} (t) \le P_{gen,rated}$$

Equations (3)–(4) is lithium bromide unit generating thermal power and cooling power function, respectively. *H*_*li,h*_(*t*) and *C*_*li,c*_(*t*) represents thermal power and cooling power output of lithium bromide unit at time step *t*, respectively. ${\eta }_{gen,h}$ represents that gas turbine’s efficiency of gas converting to waste thermal energy. ${COP}_{li,h}$ and ${COP}_{li,c}$ represents lithium bromide unit’s heating efficiency and refrigeration efficiency, respectively. *P*_*li*_(*t*) represents the consumed electricity of lithium bromide. ${\eta }_{p,li}$ represents the amount of electricity consumed to generate 1 kWh thermal energy. *S*_*li,h*_(*t*) and *S*_*li,c*_(*t*) represents refrigeration state and heating state, respectively. *H*_*li,rated*_ is the rated power of lithium bromide unit.

3 $$H_{li,h} (t) = \eta_{gen,h} \cdot COP_{li,h} \cdot \lambda_{gas} \cdot c_{gen} (t)/\Delta t$$

4 $$C_{li,c} (t) = \eta_{gen,h} \cdot COP_{li,c} \cdot \lambda_{gas} \cdot c_{gen} (t)/\Delta t$$

5 $$P_{li} (t) = \eta_{p,li} \cdot (H_{li,h} (t) + C_{li,c} (t))$$

6 $${0} \le H_{li,h} (t) \le S_{li,h} (t) \cdot H_{li,rated}$$

7 $${0} \le C_{li,c} (t) \le S_{li,c} (t) \cdot H_{li,rated}$$

8 $$S_{li,c} (t){ + }S_{li,h} (t) \le 1$$

### Boiler

The boiler can convert nature gas to thermal energy. Eq. (9) represents the calculation method of boiler output, which is equal to the product of thermal generation efficiency of boiler, calorific value of natural gas and consumed natural gas (*n*_*hw*_(*t*)). *H*_*hw*_(*t*) is boiler power output at time step *t*. *H*_*hw,rated*_ is the rated power of boiler.

9 $$H_{hw} (t) = \eta_{hw} \cdot \lambda_{gas} \cdot n_{hw} (t)/\Delta t$$

10 $$0 \le H_{hw} (t) \le H_{hw,rated}$$

### Centrifuge

Centrifuge can convert electric energy to cooling energy. Eq. (11) is the cooling energy output of centrifuge function, in which ${\eta }_{cen}$ is the efficiency of electricity converting to cooling energy. *P*_*cen*_(*t*) is electric power consumed by centrifuge. *C*_*cen*_(*t*) is centrifuge power output at time step *t*. *C*_*cen,rated*_ is the rated power of centrifuge.

11 $$C_{cen} (t) = \eta_{cen} \cdot P_{cen} (t)$$

12 $${0} \le C_{cen} (t) \le C_{cen,rated}$$

### Electric energy storage

The constraints of electric energy storage include the balance constraint, upper and lower limits of battery capacity, constraints on charging and discharging power, constraints on charging and discharging state.

13 $$E_{ba} (t) = (1 - \beta )E_{ba} (t - 1) + P_{ba,c} (t) \cdot \eta_{c} - P_{ba,dis} (t)$$

14 $$0 \le P_{ba,c} (t) \le S_{ba,c} (t) \cdot P_{ba,\max }$$

15 $$0 \le P_{ba,dis} (t) \le S_{ba,dis} (t) \cdot P_{ba,\max }$$

16 $$S_{ba,c} (t) + S_{ba,dis} (t) \le 1$$

17 $$E_{ba,\min } \le E_{ba} (t) \le E_{ba,\max }$$

where *E*_*ba*_(*t*) is the battery capacity at time step *t*. $\beta$ is loss factor of battery. ${\eta }_{c}$ is charging efficiency of battery. *P*_*ba,c*_(*t*) and *P*_*ba,dis*_(*t*) represents charging power and discharging power of battery at time step *t*, respectively. *S*_*ba,c*_(*t*) and *S*_*ba,dis*_(*t*) represents charging state and discharging state at time step *t*, respectively. *P*_*ba,max*_ is the maximum charging and discharging power. *E*_*ba,min*_ and *E*_*ba,max*_ represents upper and lower limits of battery capacity, respectively.

### Thermal energy storage

The general model of generalized energy storage system is adopted to deal with the thermal energy storage equipment in this paper. Therefore, the model of thermal energy storage is basically the same as that of electric energy storage and there is no more detailed description.

18 $$E_{sh} (t) = (1 - \gamma )E_{sh} (t - 1) + H_{sh,c} (t) \cdot \eta_{shc} - H_{sh,dis} (t)$$

19 $$0 \le H_{sh,c} (t) \le S_{sh,c} (t) \cdot H_{sh,\max }$$

20 $$0 \le H_{sh,dis} (t) \le S_{sh,dis} (t) \cdot H_{sh,\max }$$

21 $$S_{sh,c} (t) + S_{sh,dis} (t) \le 1$$

22 $$E_{sh,\min } \le E_{sh} (t) \le E_{sh,\max }$$

### External grid

MEMG is connected to the external grid, whose energy exchange range is constrained as follows:

23 $$P\_\min < P_{gird} (t) < P\_\max$$

where *P_max* and *P_min* is upper and lower limit of purchased power, respectively.

### Power balance

Equations (24), (26) and (27) represents electric power balance, thermal power balance and cooling power balance, respectively.

24 $$P(t) + P_{cen} (t) + P_{ba,c} (t) + P_{li} (t) = P_{pv} (t) + P_{gen} (t) + P_{ba,dis} (t) \cdot \eta_{dis} + P_{grid} (t)$$

25 $$P(t) = P_{bl} (t) + P_{su,cl} (t) + P_{su,bo} (t) + P_{ev} (t)$$

26 $$H(t) + H_{sh,c} (t) = H_{hw} (t) + H_{li,h} (t) + H_{sh,dis} (t) \cdot \eta_{bat,dis}$$

27 $$C_{air} (t) = C_{cen} (t) + C_{li,c} (t)$$

*P*(*t*) is electric load power at *t*^*th*^ hour. ${\eta }_{dis}$ is discharging efficiency of battery. *P*_*grid*_(*t*) represents the electricity purchased from external grid. *P*_*bl*_(*t*) is the base electric load of MEMG at *t*^*th*^ hour. *P*_*su,cl*_(*t*) and *P*_*su,bo*_(*t*) represents washing machine load and dishwasher load of MEMG at *t*^*th*^ hour, respectively, both of which belong to shiftable but uninterruptible electrical load and can be calculated by Eq. (40). *H*_*sh,c*_(*t*) and *H*_*sh,dis*_(*t*) represents input thermal power and output thermal power of thermal energy storage at *t*^*th*^ hour, respectively.

## Low-carbon economic dispatch model of MEMG

**Figure 3:**
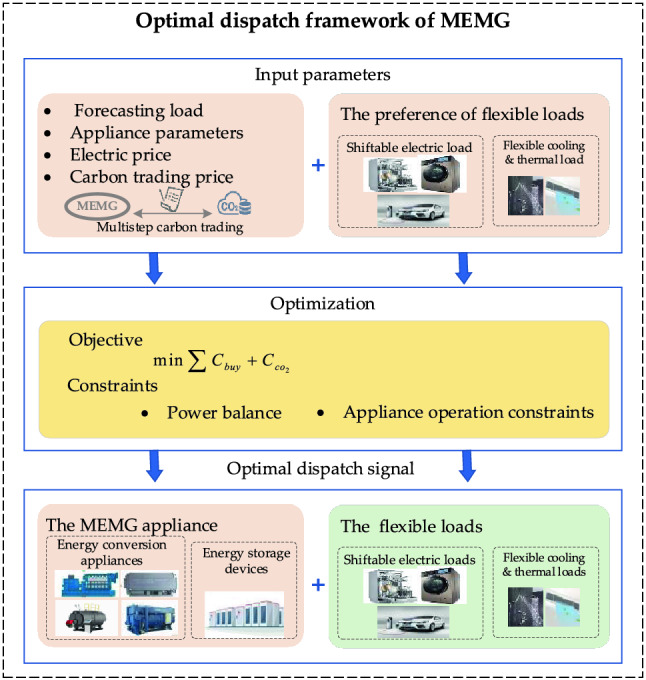
The proposed optimal dispatch framework of MEMG.

The proposed optimal dispatch framework of MEGM is shown in Fig. 3. The low-carbon economic dispatch model optimizes MEMG day-ahead operation for 24 h a day, considering multistep carbon trading and IDR. The objective function is to minimize the sum of purchased energy cost and carbon trading cost, which can be expressed as Eq. (28).

28 $$\min \sum {C_{buy} + C_{{co_{2} }} }$$

29 $$C_{buy} = \sum\limits_{t = 1}^{24} {P_{gird} (t)} .p_{buy} (t) + (n_{cen} (t) + n_{hw} (t)).p_{n}$$

where *C*_*buy*_ is the purchased energy cost including purchased electricity cost and nature gas cost, which is shown in Eq. (29). ${C}_{{co}_{2}}$ is the carbon trading cost, which is calculated by multistep carbon trading cost model shown in the next section (Multistep carbon trading cost model). *p*_*buy*_(*t*) represents the electricity price, which is a time-of-use (TOU) price, and *p*_*n*_ is the gas price.

### Multistep carbon trading cost model

For the power industry, the initial carbon emission share is generally allocated by a free way^11^. The initial free carbon emission share is related to the power generation of the system, and the excess or insufficient part can be traded. The electricity purchased from external grid is assumed that it is generated by thermal power unit in this paper. In MEMG, only external grid and gas turbine generate electricity while cause the carbon emission, therefore, the free carbon emission share of MEMG is determined by the electricity purchased from external grid and generated by gas turbine, which can be expressed as Eq. (30).

30 $$e_{f} = \delta \sum\limits_{t = 1}^{T} {(P_{grid} (t) + } P_{gen} (t))/1000$$

where *e*_*f*_ is the free carbon emission share. $\delta$ is emission share per unit of electricity, which is equal to the weighted average value of marginal emission factor of regional electricity and marginal capacity factor. It is equal to 0.572 kg/kWh^15^.

In MEMG, only gas turbine and boiler use nature gas to generate energy. Consequently, there are three carbon emission sources: electricity purchased from external grid, gas turbine and boiler. The carbon emission can be expressed as^16^:

31 $$e_{p} = a_{1} \sum\limits_{t = 1}^{T} {P_{grid} (t)} /1000 + a_{2} \sum\limits_{t = 1}^{T} {(n_{hw} (t) + n_{gen} (t))}$$

where *a*_1_ is equivalent emission coefficient of electricity purchased from grid, and it is equal to 0.972 kg/kWh. *a*_2_ is the equivalent emission coefficient of consuming natural gas, which is equal to 2.3131 × 10^–3^ t/m^3^.

**Figure 4:**
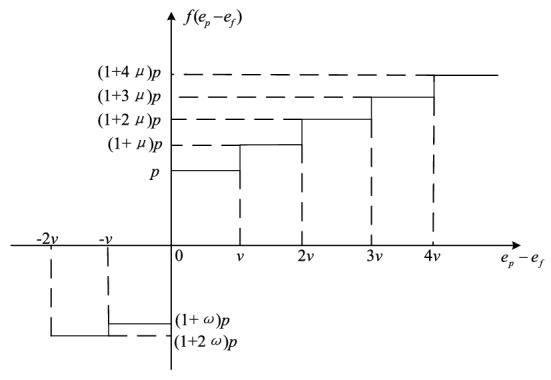
The relationship between carbon trading price and carbon trading volume.

In order to further control the total amount of carbon emission, this paper constructs a multistep carbon trading cost calculation model. Figure 4 shows the relationship between carbon trading price and carbon trading volume. A number of emission ranges are set based on the free carbon emission share.

The multistep carbon trading cost model is showed in Eq. (32). *p* is the market price for carbon trading. **ω** represents the incentive coefficient when the carbon emission is less than the free carbon emission share. $\mu$ represents the growth rate of carbon trading price in each ladder. *v* represents the interval length of carbon emissions.

32 $$C_{{co_{2} }} = \left\{ {\begin{array}{*{20}c} { - p(1 + \omega )v - p(1 + 2\omega )(e_{f} - e_{p} - v),e_{p} < e_{f} - v} \\ { - p(1 + \omega )(e_{f} - e_{p} ),e_{f} - v < e_{p} < e_{f} } \\ {p(e_{p} - e_{f} ),e_{f} < e_{p} < e_{f} + v} \\ {pv + p(1 + \mu )(e_{p} - e_{f} - v),e_{f} + v < e_{p} < e_{f} + 2v} \\ {p(2 + \mu )v + p(1{ + 2}\mu )(e_{p} - e_{f} - 2v),e_{f} + 2v < e_{p} < e_{f} + 3v} \\ {p(3 + {3}\mu )v + p(1{ + 3}\mu )(e_{p} - e_{f} - 3v),e_{f} + 3v < e_{p} < e_{f} + 4v} \\ {p(4 + {6}\mu )v + p(1{ + 4}\mu )(e_{p} - e_{f} - 4v),e_{f} + 4v < e_{p} } \\ \end{array} } \right.$$

According to Fig. 4 and Eq. (32), it is seen that when the carbon emissions are less than the free carbon emissions share, ${C}_{{co}_{2}}$ is negative. It means that the MEMG can sell excess carbon emission share in the carbon trading market and obtain certain subsidies. The less the carbon emission is, the more expensive the carbon trading price is. When the carbon emissions are greater than the free carbon emissions share, ${C}_{{co}_{2}}$ is positive. It indicates that the MEMG need to buy carbon emission rights in the carbon trading market. The larger the carbon emission is, the more expensive the carbon trading price is.

### IDR model

IDR includes demand response of electrical load, thermal load and cooling load. In this paper, the electrical load is divided into three types: 1) basic electrical load, 2) shiftable and interruptible electrical load, and 3) shiftable but uninterruptible electrical load. Thermal load is classified into basic thermal load and flexible thermal load. Cooling load is comprised of basic cooling load and flexible cooling load.

- Shiftable and interruptible electrical load
  The shiftable and interruptible electrical load considered in this paper is electric vehicle. As long as the electric vehicle (EV) capacity reaches the expected value at the departure time, the electricity demand of the user can be satisfied. Therefore, the charging process can be interrupted and the charging time can also be shifted. The model can be expressed as:
  33 $$0 \le P_{ev}^{i} (t) \le P_{c,\max } ,\forall t \in [t_{a} ,t_{g} ]$$
  34 $$P_{ev}^{i} (t) = 0,\forall t \notin [t_{a} ,t_{g} ]$$
  35 $$E_{ev}^{i} (t) = (1 - \alpha ) \cdot E_{ev}^{i} (t - 1) + P_{ev}^{i} (t) \cdot \eta_{ev}$$
  36 $$E_{\min } \le E_{ev}^{i} (t) \le E_{\max }$$
  37 $$E_{ev} (t_{g} ) = E_{\exp }$$
  where ${P}_{ev}^{i}(t)$ represents the EV charging load of user *i*. *t*_*a*_ and *t*_*g*_ represents the time when the user arrives at home and leaves home, respectively. ${E}_{ev}^{i}(t)$ is the EV electric capacity. $\alpha$ is loss factor and ${\eta }_{ev}$ is charging efficiency. Eq. (33) restricts the EV charging power. Eq. (35) represents the capacity of EV at time step *t* is decided by the capacity of EV at time step *t*-1 and EV charging electricity at time step *t*. Eq. (36) constraints the EV capacity. Eq. (37) represents that the EV capacity must reach the expected value at the departure time.
- Shiftable but uninterruptible electrical load
  This paper assumes that the working duration of the user's transferable and uninterruptible device *q* is denoted as *T*_*wo*_, and the working range is [*t*_*s*_, *t*_*e*_], and there are *n* users. Since the load is uninterruptible, the allowable working time is divided into *t*_*e*_-*t*_*s*_-*T*_*wo*_ + 2 periods. The divided period is represented by the vector ***f***, and the value of the vector element represents the number of user that the device works during this period. Therefore, the shiftable but uninterruptible electrical load model are expressed as Eqs. (38), (39) and (40).
  38 $$\sum\limits_{i = 1}^{{t_{e} - t_{s} - T + {2}}} {f_{i} = n}$$
  39 $$f_{i} \in N^{ + }$$
  40 $$P_{su,b} (t) = \left\{ \begin{gathered} 0, \, t \notin [t_{s} ,t_{e} ] \hfill \\ \sum\limits_{i = 1}^{{t - t_{s} + 1}} {f_{i} .P_{b,rated} } , \, t_{s} \le t \le t_{s} + T_{wo} - 1 \, \hfill \\ \sum\limits_{{i = t - t_{s} - T_{wo} + 2}}^{{t - t_{s} + 1}} {f_{i} .P_{b,rated} } , \, t_{s} + T_{wo} - 1 \le t \le t_{e} - T_{wo} + 1 \hfill \\ \sum\limits_{{i = t - t_{s} - T{}_{wo} + 2}}^{{t_{e} - t_{s} - T_{wo} + 2}} {f_{i} .P_{b,rated} } , \, t_{e} - T_{wo} + 1 \le t \le t_{e} \hfill \\ \end{gathered} \right.$$
  where *P*_*su,b*_(*t*) is electric power of the device *b* at time step *t* and *P*_*b,rated*_ is rated power of the device *b*. *f*_*i*_ represents element, *i* in vector ***f***.
- Flexible thermal and cooling load

The flexible thermal load considered in this paper is the hot water load. The user has an acceptable range for water temperature, which can be expressed as $[{T}_{h,min},{T}_{h,max}]$. Therefore, the thermal load power to maintain the water temperature should also be expressed as an interval^17^:

41 $$H_{hw,\min } = C_{w} p_{w} V_{cold} (t)[T_{h,\min } - T_{ini} ]/\Delta t$$

42 $$H_{hw,\max } = C_{w} p_{w} V_{cold} (t)[T_{h,\max } - T_{ini} ]/\Delta t$$

43 $$H_{hw,\min } \le H(t) \le H_{hw,\max }$$

In Eq. (41), *C*_*w*_ is specific heat capacity of water, which is equal to 1.1667 × 10^–3^ kWh/kg.℃. *p*_*w*_ is the density of water, which is equal to 1000 kg/m^3^. *V*_*cold*_(*t*) is the volume of water used by users at time step *t*, and $\Delta t$ is the time step. *H*(*t*) is thermal power at time step *t*.

The flexible cooling load considered in this paper is the air conditioner load. The theory of flexible cooling load is basically the same as that of thermal load, so the flexible cooling load model can be expressed as:

44 $$C_{air,\min } = [T_{out} (t) - T_{air,\min } ]/R$$

45 $$C_{air,\max } = [T_{out} (t) - T_{air,\max } ]/R$$

46 $$C_{air,\min } \le C_{air} (t) \le C_{air,\max }$$

where *T*_*out*_(*t*) represents the outside temperature at time step *t*. *R* represents the building thermal resistance, which is equal to 18 ℃/kW. *T*_*air,min*_ and *T*_*air,max*_ represents the maximum and minimum temperature that satisfy user’s demand, respectively. *C*_*air*_(*t*) is cooling power at time step *t*.

## Simulation and results

As described in Sect. 2, the proposed model is conducted on a large-scale energy hub where there are 2000 residents, over a daily time horizon with time interval of one hour. The parameters of shiftable but uninterruptible load are shown in Table 1. ^18^. The parameters of energy conversion appliances and storage devices are shown in Table 2. ^16^. As for EV, the users arrive home at 18:00 and leave home at 8:00, and the surplus electricity at 18:00 is 1kWh, and the expected electricity at 8:00 is 24kWh, and the loss factor is 0.01, and the charging efficiency is 0.95, and the maximum charging power is 3.6 kW. Flexible thermal load considered in this paper is hot water load, and flexible cooling load is air conditioner load. The acceptable range for hot water temperature $$[{T}_{h,min},{T}_{h,max}]$$ is [65,75]. The acceptable range for indoor temperature $$[{T}_{air,min},{T}_{air,max}]$$ is [22,26]. In multistep carbon trading cost model, *p* is 44 $/t, $$\omega$$ is 0.2, *v* is 30 t and $$\mu$$ is 0.25. Electricity price is shown in Table 3.

**Table 1:** The parameters of shiftable but uninterruptible load.

Load	Rated power (kW)	Working duration (h)	Working range
Washing machine	0.6	2	[9, 18]
Dishwasher	0.8	2	[19, 24]

**Table 2:** The parameters of energy conversion appliances.

Appliances	Parameters	Value
Gas turbine	Rated power	1000*3 kW
	Gas-heat conversion efficiency	0.45
	Gas-electric conversion efficiency	0.35
Lithium bromide unit	Rated power	1000*3 kW
	Refrigeration efficiency	1.2
	Heating efficiency	0.8
	Consuming electricity	0.02
Boiler	Rated power	1000*3 kW
	Gas-heat conversion efficiency	0.95
Centrifuge	Rated power	1750*3 kW
	Electric-cooling conversion efficiency	3.0
Electric energy storage	Maximum capacity	2700 kWh
	Minimum capacity	300 kWh
	Maximum input or output power	500 kW
	Charging or discharging efficiency	0.96
	Loss factor	0.01
Thermal energy storage	Maximum capacity	1680 kWh
	Minimum capacity	200 kWh
	Maximum input or output power	700 kW
	Charging or discharging efficiency	0.96
	Loss factor	0.02
External grid	Minimum exchange power	0 kW
	Maximum exchange power	10,000 kW

**Table 3:** Electricity price.

Period	7PM–10PM	8AM–11AM 3PM–7PM	7AM–8AM 11AM–3PM 10PM–11PM	11PM–7AM
Price ($/kWh)	0.13897	0.12325	0.09967	0.06823

**Figure 5:**
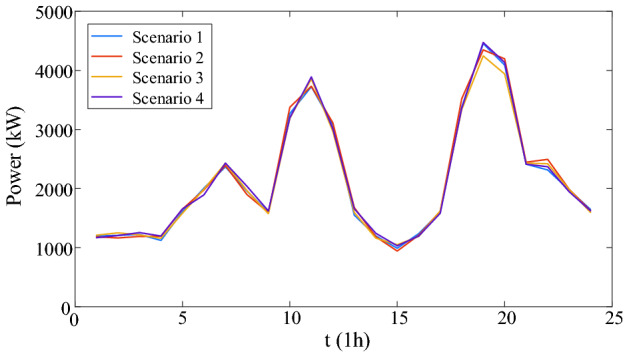
The scenario set of basic electrical load.

**Figure 6:**
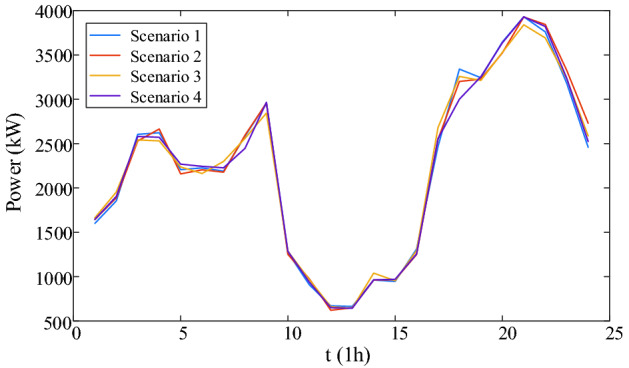
The scenario set of thermal load.

**Figure 7:**
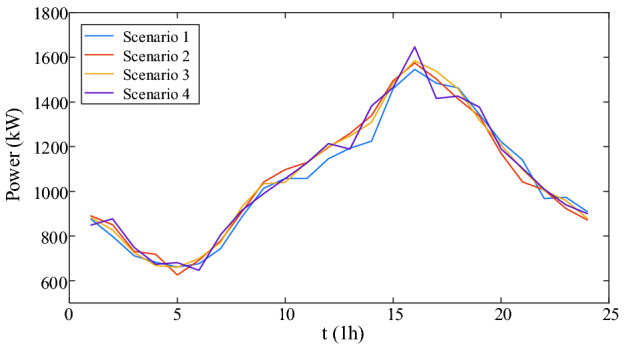
The scenario set of cooling load.

Considering the uncertainty of load, scenario generation method based on sequential Monte Carlo simulation^19^ is used in this paper to generate multiple scenarios. The basic electric load, thermal load and cooling load of four scenarios generated by the sequential Monte Carlo simulation are shown in Figs. 5, 6, 7.

### Case study

According to the loads of four scenarios, four deterministic optimal dispatches are carried out. The carbon emission and cost of four scenarios are shown in Table 4.

**Table 4:** Carbon and cost of four scenarios.

Scenario	Carbon emission (t)	Carbon trading cost ($)	Purchased energy cost ($)	Total cost ($)
Scenario 1	95.7782	1351.45	12,229.53	13,580.98
Scenario 2	96.1891	1356.32	12,314.10	13,670.43
Scenario 3	95.5079	1341.86	12,204.38	13,546.24
Scenario 4	96.1796	1358.68	12,276.53	13,635.21

In order to evaluate the proposed model, four case studies are considered according to Table 5. The simulation results are then compared and discussed. To be more precisely, in case 1, IDR are not considered. Multistep carbon trading is not considered. the carbon trading model of^9^ is applied to calculate the carbon trading cost, where the carbon trading price is constant. In case 2, IDR is not considered but multistep carbon trading is considered. In case 3, the residents are equipped with smart meter to control shiftable electrical equipment and collect customers’ real-time data. Therefore, the basic requirement for conducting IDR program by determined responsive loads are enabled. Multistep carbon trading is not considered. In case 4, both IDR and multistep carbon trading are considered. The load data of scenario 1 is used to analyze the results of four cases.

**Table 5:** Summary of case studies.

Case	IDR	Multistep carbon trading
Case 1	×	×
Case 2	×	√
Case 3	√	×
Case 4	√	√

In Table 6, It is seen that carbon emission and total cost is maximum in case 1. Comparing case 1 with case 3, both carbon emission and total cost decreased, which shows that considering IDR reduce not only total cost but also carbon emission. Comparing case 4 with case 2, it is seen that carbon emission and total cost of case 4 are less than that of case 2. From above results and analysis, it is illustrated that the low-carbon economic dispatch model of this paper can give consideration to economy and environmental protection.

**Table 6:** Carbon emission and cost comparison of four cases.

Case	Carbon emission (t)	Carbon trading cost ($)	Purchased energy cost ($)	Total cost ($)
Case 1	106.2759	1781.23	15,033.66	16,814.90
Case 2	97.9680	1428.79	15,419.43	16,848.22
Case 3	103.6621	1699.80	11,865.30	13,565.10
Case 4	95.7782	1351.45	12,229.53	13,580.98

**Figure 8:**
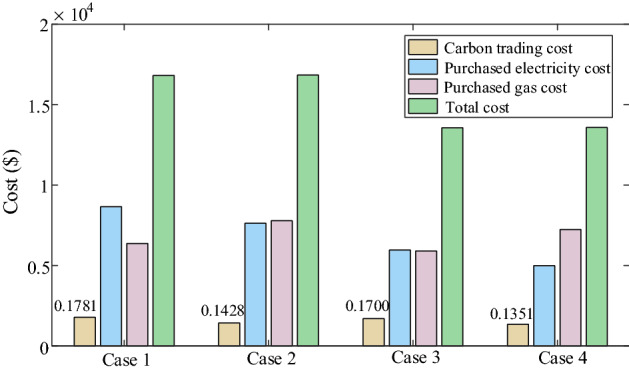
The specific cost of four cases.

Judging from Fig. 8, it is seen that considering multistep carbon trading leads to purchased electricity cost decreasing and purchased nature gas cost increasing. It means that when the carbon emissions are limited, the energy required by MEMG will shift from electricity to natural gas, and the cost of energy will increase. Combining Table 6 and Fig. 8, multistep carbon trading decreases carbon emission and decreases the carbon trading cos t in that multistep carbon trading can constraint carbon emission by multistep carbon trading price.

### Impact of IDR on MEMG operation

**Figure 9:**
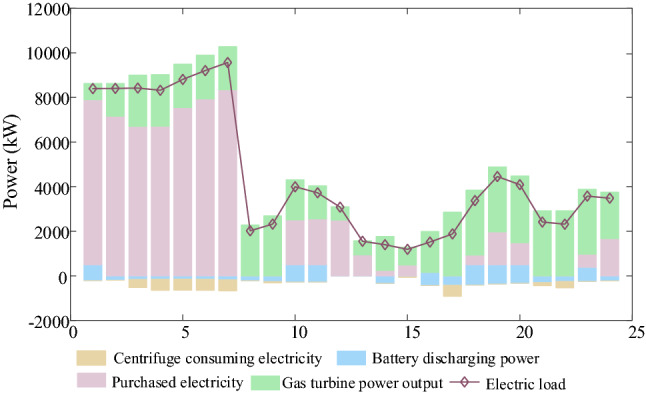
Electricity supply and demand of case 4.

**Figure 10:**
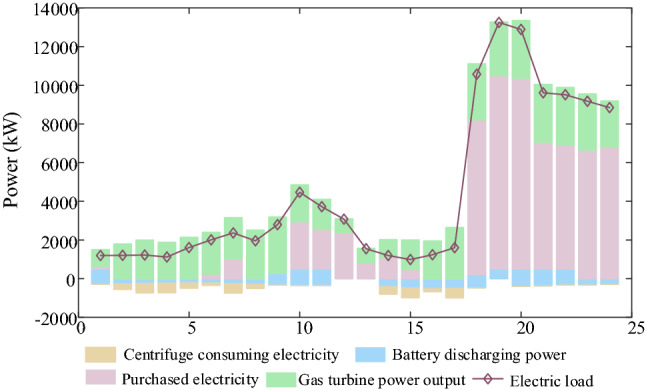
Electricity supply and demand of case 2.

**Figure 11:**
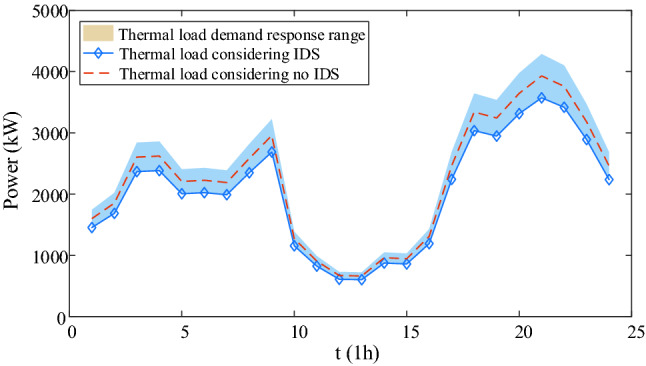
Thermal load comparison considering IDR and no IDR.

**Figure 12:**
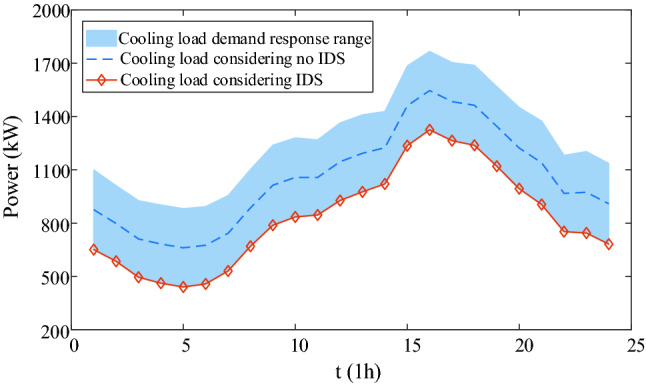
Cooling load comparison considering IDR and no IDR.

IDR are shown in Figs. 9, 10, 11, 12. Comparing Figs. 9 and 10, it is seen that the peak electric load is concentrated at 18:00–24:00 without IDR, but the electricity price is expensive at this time. However, considering IDR, the shiftable loads is shifted at 1:00–7:00 when the electricity price is cheap, which decreases the energy cost greatly. In Figs. 11 and 12, it is seen that the blue area is thermal load and cooling load comfortable adjustment range considering demand response. Considering IDR, it is obvious that the load reduction has been completed within the appropriate range, which can reduce the energy consumption. Consequently, the energy cost and carbon emission are reduced. Therefore, considering IDR can decrease the cost and carbon emission by adjusting the shiftable electricity load into the period that the electricity price is cheap and reducing the thermal and cooling load base on not affecting the user’s comfort level.

### Sensitivity analysis

**Figure 13:**
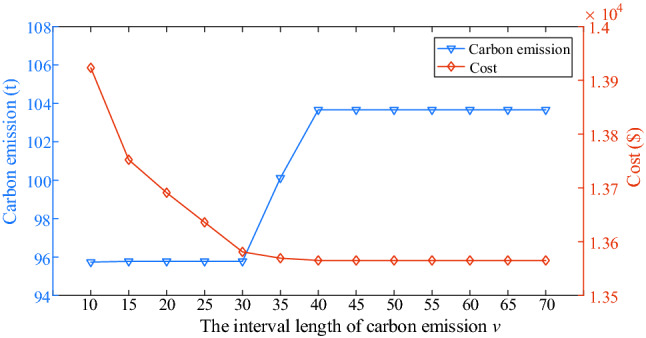
Variation trend of carbon emissions and costs at different *v* in case 4.

In Fig. 4, it is seen that different interval lengths of carbon emission (*v*) lea d to different results. To study the impact of the interval length of carbon emission on MEMG operation and get an optimal parameter *v*, sensitivity analysis is carried out. Figure 13 shows the variation trend of carbon emissions and cost at different *v* in case 4. It is seen that the carbon emission increases greatly when *v* is equal to 35 t comparing with that when *v* is equal to 30 t. Meanwhile, the cost declines when *v* is less than 30 t and the cost is almost changeless when *v* is more than 30 t. The reason is that the less *v* is, the more stringent the constraint of multistep carbon trading model on carbon emission. Therefore, when *v* is less than 30 t, the change of cost is obvious. However, with the increment of *v*, the carbon emission reaches at a critical value, so the cost is almost changeless. Additionally, that *v* is equal to 30 t is optimal considering both carbon emission and cost from Fig. 13.

**Figure 14:**
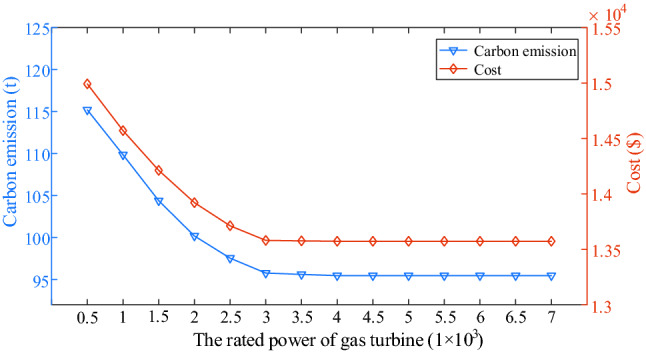
Variation trend of carbon emissions and costs at different rated power of gas turbine in case 4.

To study the impact of gas turbine on carbon emission and cost, the rated power of gas turbine is changed to obtain the MEMG operation result. In Fig. 14, it is obvious that when the rated power is less than 3 × 10^3^ kW, the more rated power is, the less carbon emission and cost are, which indicates the advantages of gas turbine on environment and economy. However, as the rated power continues increasing, the cost and carbon emission remain nearly constant. The reason is that the purchased electricity is much more than gas turbine power output during the peak consuming electricity period considering IDR. Additionally, the installed rated power of gas turbine is equal to 3 × 10^3^ kW is enough to satisfy the MEMG optimal operation due to Fig. 14.

## Conclusion

In this paper, a low-carbon economic dispatch model of MEMG considering IDR and multistep carbon trading is proposed to give consideration to economy and environmental protection. The optimal results of multiple scenarios considering load uncertainty are shown and the results of four cases are compared. To considering the influence of the parameter *v* (the interval length of carbon emission) and the rated power of gas turbine, sensitivity analysis is carried out.

The main claims are derived: (1) The low-carbon economic dispatch model has a stringent control effect on carbon emissions, while taking into account the overall economy of MEMG. (2) Considering IDR can decrease the cost and carbon emission by shifting the shiftable electricity load to the period when the electricity price is cheap and reducing the thermal and cooling load base on not affecting the user’s comfort level. (3) That the parameter *v* (the interval length of carbon emission) in multistep carbon trading model is equal to 30 t is optimal in this paper. (4) The installed rated power of gas turbine is equal to 3 × 10^3^ kW is enough to satisfy the MEMG optimal operation. In conclusion, the proposed low-carbon economic dispatch model in this paper can decrease the carbon emission while reducing operation cost, which is benefit for environment and economic operation.

As future work, with the launch of the national carbon trading system, the government's carbon constraint on the power industry will become more stringent. Therefore, how to set the most appropriate interval length and price increase range for the low-carbon economic scheduling model is a meaningful research direction.
